# Antioxidant Properties and Hyphenated HPLC-PDA-MS Profiling of Chilean *Pica* Mango Fruits (*Mangifera indica* L. Cv. piqueño)

**DOI:** 10.3390/molecules19010438

**Published:** 2013-12-31

**Authors:** Javier E. Ramirez, Ricardo Zambrano, Beatriz Sepúlveda, Mario J. Simirgiotis

**Affiliations:** 1 Laboratorio de Productos Naturales, Facultad de Ciencias Básicas, Universidad de Antofagasta, Avenida Universidad de Antofagasta 02800, Antofagasta 1240000, Chile; E-Mails: je.ramirez.007@gmail.com (J.E.R.); soulmaster_rz@hotmail.com (R.Z.); 2 Departamento de Ciencias Químicas, Universidad Andrés Bello, Campus Viña del Mar, Los Fresnos N 52, Viña del Mar 2520000, Chile; E-Mail: bsepulveda@uc.cl

**Keywords:** *Pica* mango fruits, *Tommy Atkins*, antioxidant capacities, HPLC-PDA-ESI-ToF-MS, local Chilean plants, nutraceuticals, mangiferin, xanthones, flavonoids, phenolic acids

## Abstract

Antioxidant capacities and polyphenolic contents of two mango cultivars from northern Chile, one of them endemic of an oasis in the Atacama Desert, were compared for the first time. Twenty one phenolic compounds were detected in peel and pulp of mango fruits varieties *Pica* and *Tommy Atkins* by HPLC-PDA-MS and tentatively characterized. Eighteen compounds were present in *Pica* pulp (ppu), 13 in *Pica* peel (ppe) 11 in *Tommy Atkins* pulp (tpu) and 12 in *Tommy Atkins* peel (tpe). Three procyanidin dimers (peaks **6**, **9** and **10**), seven acid derivatives (peaks **1**–**4**, **11**, **20** and **21**) and four xanthones were identified, mainly mangiferin (peak **12**) and mangiferin gallate, (peak **7**), which were present in both peel and pulp of the two studied species from northern Chile. Homomangiferin (peak **13**) was also present in both fruit pulps and dimethylmangiferin (peak **14**) was present only in *Tommy* pulp. *Pica* fruits showed better antioxidant capacities and higher polyphenolic content (73.76/32.23 µg/mL in the DPPH assay and 32.49/72.01 mg GAE/100 g fresh material in the TPC assay, for edible pulp and peel, respectively) than *Tommy Atkins* fruits (127.22/46.39 µg/mL in the DPPH assay and 25.03/72.01 mg GAE/100 g fresh material in the TPC assay for pulp and peel, respectively). The peel of *Pica* mangoes showed also the highest content of phenolics (66.02 mg/100 g FW) measured by HPLC-PDA. The HPLC generated fingerprint can be used to authenticate *Pica* mango fruits and *Pica* mango food products.

## 1. Introduction

**Figure 1:**
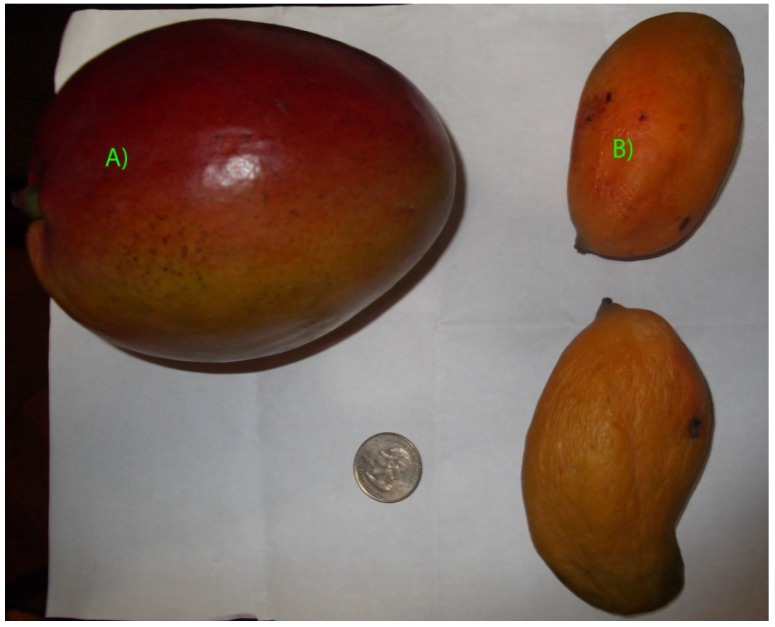
Photograph of (**A**) ripe *Tommy Atkins* Mango Fruit and (**B**) *Pica* Mango Fruits.

*San José de Pica* (from aboriginal quechua language: piqai, flower in the sand) is a small town and oasis in a remote part of the Atacama Desert located in the region of Tarapacá (I region of Chile), 114 km southeast of the city of Iquique. *Pica* has a lush greenery and thriving agriculture due to underground water sources surfacing in the middle of the Atacama Desert. Due to its water supply *Pica* has been inhabited for millennia, and it was a vital point on the Inca road system south from Peru. The *Oasis of Pica* is well known for the plentiful amounts of typical mango fruits and lemons that grow there, particularly the *Limon de Pica* (Pica lemon) and *Mango de Pica* (Pica mango) the first one a small and tart lemon that is famous throughout Chile. The mango (*Mangifera indica* L.) is a fleshy stone fruit belonging to the genus *Mangifera*, consisting of numerous tropical fruiting trees in the flowering family of *Anacardiaceae* in the order *Rutales*. Mango is one of the most important tropical fruits worldwide [1], it is a fruit with high nutritional value and unique flavors and taste, considered a good source of antioxidants, including vitamin C [2], and different xanthones [3] carotenoids [4], flavonoids [5,6], benzophenones [5], phenolic acids [7,8] and tannins [9,10] with attributed human health benefits [11]. However, there are differences regarding the polyphenolic compounds and dietary fiber present in different varieties of *Mangifera indica* fruits [12,13] and leaves [12]. *Mango de Pica* (*Mangifera indica cv piqueño or Pica*) is a small mango fruit, six or seven times smaller than *Mango Tommy Atkins*, with an average weight of 50–100 g and a orange-yellow peel at maturity stage (Figure 1). This fruit contain considerable amounts of dietary fiber with good flavors and aroma which makes the consumers preference inclined to *Pica* mangoes in northern Chile (I and II regions of Chile). *Tommy* mangoes are bigger; the average weight is 600 g and the peel is green turning to red in maturity stage (Figure 1), however the pulp has less flavor and aroma than *Pica* mangoes. *Pica* and *Tommy* mango fruits are the two main mango varieties cultivated and consumed in northern Chile [14]. Besides the various benefits of the edible flesh the peels of mango fruits account for 15%–20% of the weight and are byproducts in the production of canned mango fruit and juices [15,16]. Indeed, mango peels showed good amounts of dietary fiber and antioxidant capacity and are considered a rich source of polyphenols, anthocyanin and carotenoids [11,13]. In this work we have analyzed *Mango de Pica* fruits (*Mangifera indica* cv piqueño, Figure 1) using HPLC with PDA and ESI-ToF-MS analyzers and made a comparison with *Mango Tommy Atkins* fruits, (*Mangifera indica* cv Tommy Atkins) harvested and consumed in northern Chile. Total phenolic content (TPC) and total flavonoid content (TFC) were compared, as well as antioxidant power measured by the bleaching of the DPPH radical, the ferric reducing antioxidant power (FRAP) and superoxide anion scavenging activity (SA), of pulp and peel of the fruits. This is the first report of phenolic constituents and HPLC analysis of *Pica* mango fruits.

## 2. Results and Discussion

### 2.1. Antioxidant Capacity and Phenolic Content of Mango Fruits from Northern Chile

**Figure 2:**
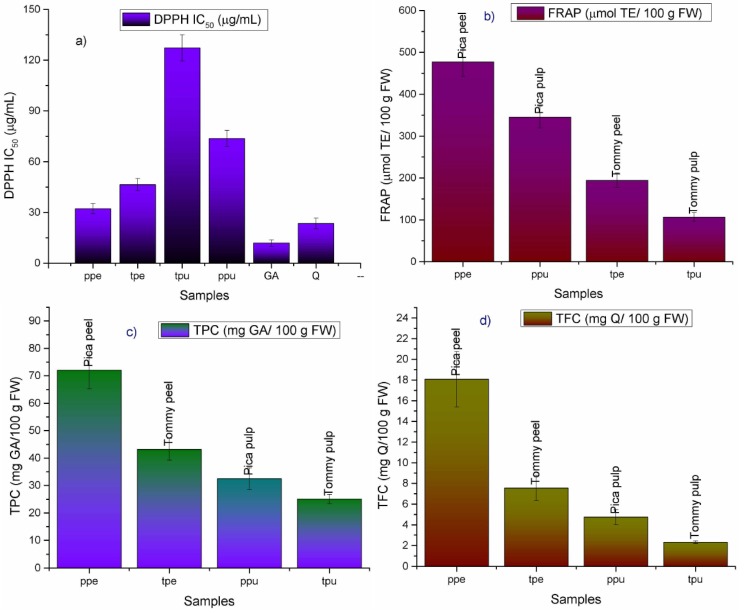
(**a**) DPPH scavenging activity; (**b**) Ferric reducing antioxidant power (FRAP); (**c**) Total Phenolic content (TPC) and (**d**) Total flavonoid content (TFC) of mango fruit extracts.

The bleaching of the DPPH radical (DPPH), total phenolic content (TPC), total flavonoid content (TFC), and ferric reducing antioxidant power (FRAP) from pulp and peel of *Tommy* and *Pica* mango fruits are shown in Figure 2. We were not able to find any HPLC analysis and identification of phenolic compounds of *Pica* mango fruits nor comparison with other mango fruits. However, the ethanolic extract of the bark from *Pica* mango trees was studied regarding antioxidant and analgesic activities and the values were compared with those of the mango varieties *Zill*, *Gloria*, *Kent*, *Sensation*, *Keitt* and *Tommy Atkins* cultivated in the same area (I region) of Chile [14]. Among those seven cultivars the ethanolic extract from *Pica* mango bark showed the highest antioxidant activity measured by the bleaching of the DPPH radical (around 96% of bleaching of the DPPH radical at 0.233 mg/mL, close to that of the positive control Trolox, with 99%) [14]. In this work methanolic extracts of peel and pulp from *Tommy* mango fruits (*Mangifera indica* L. variety Tommy Atkins) and *Pica* mango fruits (*Mangifera indica* L. variety piqueño) collected in the first region of Chile were evaluated for antioxidant power by the DPPH scavenging activity (measured as IC_50_ values) and the ferric reducing antioxidant power assay (FRAP) and the results were compared. Both fruits showed moderate to high antioxidant power, but the peel from endemic *Pica* mango fruits presented the highest activity (Figure 2). *Pica* mango fruits showed the highest DPPH scavenging activity (IC_50_ = 73.76 ± 2.08 and 32.23 ± 2.99 µg/mL, for pulp and peel, respectively, Figure 2) and higher ferric reducing antioxidant power (194.25 ± 17.43 and 477.23 ± 34.61 µmol TE (Trolox equivalents)/100 g fresh weight, for pulp and peel, respectively, Figure 2) than *Tommy* fruits peel and pulp power (127.22/46.39 µg/mL, in the DPPH assay and 106.71/345.49 µmol TE (Trolox equivalents)/100 g FW, for pulp and peel, respectively, Figure 2). The pulp of *Pica* mango fruits showed total phenolic content of 32.49 ± 3.91 mg GAE (gallic acid equivalents) per 100 g fresh material. This value is 1.29 times higher than the content in *Tommy* fruits (25.03 ± 1.72 mg GAE/100 g fresh material), collected in the same location. The peels showed similar trend (Figure 2). For *Pica* mangoes the total phenolic content of the peel was 2.2 times higher (72.01 ± 2.78 mg GAE/100 g fresh material), than its pulp, while for *Tommy* mangoes peel was 1.72 times higher (43.17 ± 3.95 mg GAE/100 g fresh material), than its pulp, which make the peels a better source of bioactive compounds. 

ppu: *Mangifera indica* L. variety *Pica* pulp extract; ppe: *Mangifera indica* L. variety *Pica* peel extract; tpu: *Mangifera indica* L. variety *Tommy Atkins* pulp extract; tpe: *Mangifera indica* L*.* variety Tommy Atkins peel extract. GA: Gallic acid, Q: Quercetin.

The content of phenolics in edible pulp of *Tommy* mangoes was close to that reported for the variety *Irwin* (20.94 ± 1.69 mg gallic acid/100 g FW) [17] and the *Tommy Atkins* varieties growing in United States (21.16 mg gallic acid/100 g FW) [18] as well as in Ecuador and Brazil (23.6 mg gallic acid/100 g FW) [19] and the variety *Haden* cultivated in Mexico (27.7 mg) [19]. However, the pulp from *Pica* mangoes showed values close to that reported for the Chinese variety *Ao* (29.45 ± 4.14 mg gallic acid/100 g FW) [17] and the variety *Kent* from Mexico (32.2 gallic acid/100 g FW) [19], while the peels from *Pica* mangoes showed values close to that of the varieties *Xiaoji* (80.50 ± 3.63 mg gallic acid/100 g FW) from China [17] and *Ataulfo* from Mexico (99.5 mg gallic acid/100 g FW) [19]. *Pica* mango fruits also showed higher values in total flavonoids (4.74 ± 0.73 mg QE (quercetin equivalents)/100 g fresh material) than Tommy mango fruits (2.32 ± 0.12 mg QE/100 g fresh material), while the highest content of flavonoids was found in *Pica* mango peels (18.07 ± 2.68 mg QE/100 g fresh material), which was close to that reported for the variety *Mallika* (18.33 ± 6.56 mg rutin/100 g fresh material) [17].

### 2.2. Identification of Phenolic Compounds in Mango Fruits by HPLC-DAD and ToF-ESI-MS/MS

**Figure 3:**
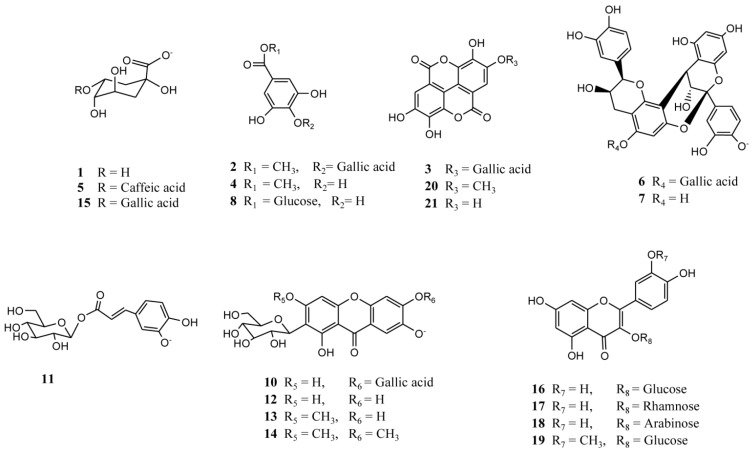
Compounds identified in two mango fruits (varieties *Pica* and *Tommy*) from Northern Chile.

**Figure 4:**
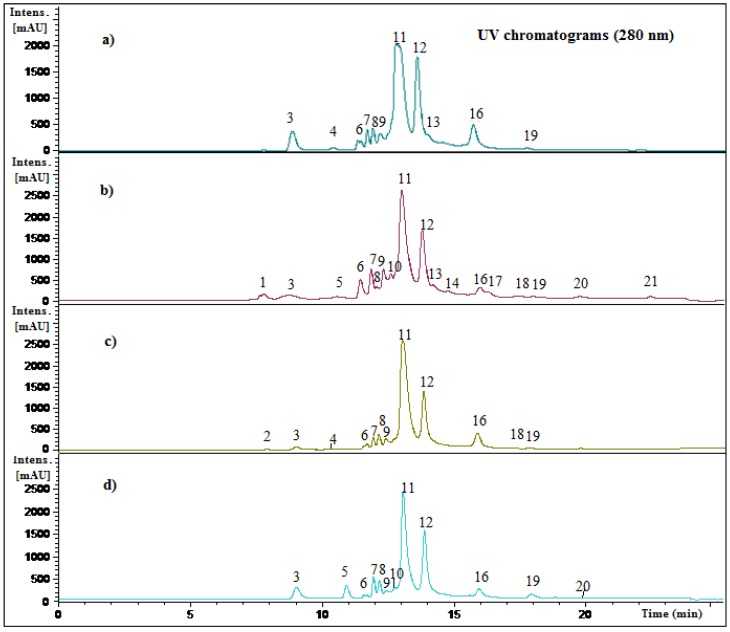
HPLC UV chromatograms at 280 nm of (**a**) *Tommy Atkins* mango fruits pulp extract, (**b**) *Pica* mango fruits pulp extract, (**c**) *Tommy Atkins* mango fruits peel extract and (**d**) *Pica* mango fruits peel extract.

In this study several compounds (Figure 3) were identified in mango fruits from northern Chile using photodiode array detection (PDA) and negative electrospray ionization-time of flight mass spectrometry (ESI-ToF-MS) in full scan mode and tandem MS/MS fragmentations. The HPLC fingerprint recorded at 280 nm of methanolic extracts from peel and pulp of both mango fruits cultivated in northern Chile is shown in Figure 4.

**Figure 5:**
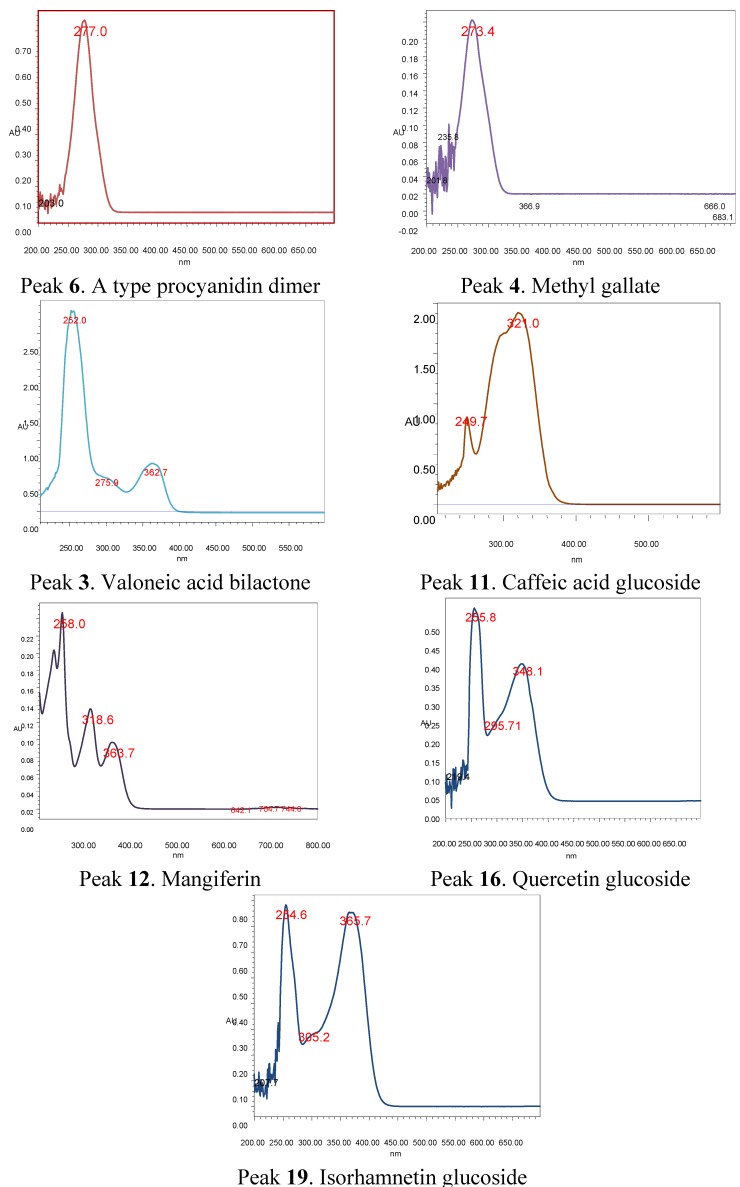
PDA spectra of compounds **3**, **4**, **6**, **11**, **12**, **16** and **19**.

Using HPLC coupled to a ToF mass analyzer the solvent flow should be less than 0.5 mL per minute and the amount of acid should be very low, since more than 0.1% of trifluoracetic (TFA) or formic acid in the solvent system as currently used in HPLC coupled to PDA detectors can damage the ToF detector. Thus, in this work we have used 0.05% formic acid in the solvent system and a flow rate of 0.4 mL/min. Both positive and negative mass conditions were employed in this work, but the acidic nature of the compounds present in the extracts (phenols) made the ions more abundant and easily detected in negative mode. It was not possible to distinguish unequivocally all detected compounds due to the lack of standard compounds. Therefore the structures of these compounds were proposed based on UV maxima (272 nm for catechin derivatives, 252, 362 nm for ellagic acid derivatives, 240, 321 nm for caffeic acid derivatives, 258, 318, 363 nm for mangiferin derivatives, 255, 354 nm for quercetin derivatives and 254, 365 nm for isorhamnetin derivatives, Figure 5) as well as fragmentation pattern thorough ESI-MS-MS experiments. In this work using tandem MS experiments the loss of 162 Daltons is indicative of hexoses (glucose or galactose, the most common sugars found in flavonoids) the loss of 146 Daltons is indicative of rhamnose, the loss of 133 Daltons is indicative of pentoses (xylose or arabinose, the most common pentoses found in natural products) [20], while the losses of 90 and 120 Daltons is indicative of C-glycoside phenolic compounds [21].

Table 1 show retention times of the peaks detected, UV maxima, molecular formula, pseudomolecular ions and MS fragmentation of all compounds detected in two cultivars of mango fruits cultivated in northern Chile plus references to the compounds, while the identification using HPLC hyphenated with PDA-ESI-ToF-MS and MS^n^ experiments of all detected and tentatively characterized compounds is explained above. In this work 21 compounds were detected and tentatively characterized, 18 in *Pica* pulp (ppu), 13 in *Pica* peel (ppe) 11 in *Tommy Atkins* pulp (tpu) and 12 in *Tommy Atkins* peel (tpe) (Table 2).

**Table 1:** Identification of phenolic compounds in mango pulp and peel by LC-PDA, LC–ESI-ToF-MS and MS/MS data.

Peak #	Rt (min)	HPLC-DAD λ max (nm)	[M-H]^−^	Formula	Other MS-MS ions (*m/z*)	Tentative identification	Reference	Species/Fruit part
1	7.8	220	191.0	C_7_H_12_O_6_	104.0	Quinic acid	[22]	ppu
2	8.0	273	335.05	C_15_H_12_O_9_	241.11, 183.03	Methyl-di-gallate ester	[22]	tpe
3	9.1	252, 275sh, 362	469.0	C_21_H_10_O_13_	169.0 (gallic acid)	Valoneic acid bilactone	[23,24]	ppu, ppe tpu, tpe
4	10.6	273	183.03	C_8_H_8_O_5_	169.1(gallic acid)	Methyl gallate	[22]	tpu, tpe
5	10.9	249, 321	353.1	C_16_H_18_O_9_	179.0 (caffeic acid)	Caffeoyl-quinic acid	[25]	ppu, ppe
6	11.7	276	727.5	C_37_H_28_O_16_	575.1 (procyanidin dimer), 405.1,284.0, 169.0 (gallic acid)	Galloyl-A-type procyanidin dimer	[26]	tpu, tpe ppu, ppe
7	12.0	277	575.12	C_30_H_23_O_12_	423.1, 285.0 (catechin), 205.1, 193.0	A-type-procyanidin dimer	[26]	ppu, ppe tpu, tpe
8	12.2	273	331.1	C_13_H_16_O_10_	169.0 (gallic acid)	Galloyl glucose	[27]	ppu, ppe tpu, tpe
9	12.5	277	559.1	C_30_H_23_O_11_	407.1, 287.0 (catechin)	Epiafzelechin-epicatechin dimer	[28]	ppu, ppe tpu, tpe
10	12.8	278, 319, 364	573.1	C_26_H_22_O_15_	421.1(Mangiferin), 169.0 (gallic acid)	Mangiferin gallate	[5]	ppu, ppe
11	13.2	249, 321	341.1	C_15_H_18_O_9_	683.6 ([2M-H]^–^), 179.6 (caffeic acid)	Caffeoyl-glucose	[25]	ppu, ppe tpu, tpe
12	14.0	258, 319, 364	421.0	C_19_H_18_O_11_	331.0 ([2M-H]^−^), 301.0 ([2M-H]^−^),	Mangiferin *	[3]	ppu, ppe tpu, tpe
13	14.3	258, 319, 364	435.1	C_20_H_19_O_11_	315.1([2M-H]^−^), 301.0, ([2M-H]^–^), 271.1	Homomangiferin	[29]	tpu, ppu
14	15.1	258, 319, 364	449.2	C_21_H_22_O_11_	315.1([2M-H]^−^), 301.0([2M-H]^−^)	Dimethylmangiferin	[30]	ppu
15	15.6	273	343.2	C_14_H_16_O_10_	191.1 (quinic acid)	Galloyl-quinic acid	[22]	ppe
16	16.0	255, 350	463.1	C_21_H_20_O_12_	301.04(quercetin), 179.0, 151.0	Quercetin-3-O-glucose *	[6]	ppu, ppe tpu, tpe
17	16.5	254, 290 sh, 351	447.1	C_21_H_20_O_11_	301.0 (quercetin), 179.0, 151.0	Quercetin-3-O-rhamnose	[6]	ppu
18	17.7	255, 293sh, 354	433.09	C_20_H_17_O_11_	301.04(quercetin), 179.0, 151.0	Quercetin-3-O-pentose	[3]	tpe, ppu
19	18.0	254, 300sh, 365	477.1	C_22_H_22_O_12_	315.1(Isorhamnetin), 300.1	Isorhamnetin-3-O-glucose *	[3]	ppu, ppe tpu, tpe
20	19.8	252, 362	315.0	C_15_H_8_O_8_	257.0, 195.03	Methyl-ellagic acid	[31]	ppu, ppe
21	22.5	252, 362	301.0	C_14_H_6_O_8_	257.0	Ellagic acid *	[5]	ppu

Species and fruit parts: *Mangifera indica* L. variety piqueño: Pulp (ppu) and peel (ppe); *Mangifera indica* L. variety Tommy Atkins: Pulp (tpu) and peel (tpe).* identified by spiked experiments with authentic standard.

#### 2.2.1. Xanthones

**Figure 6:**
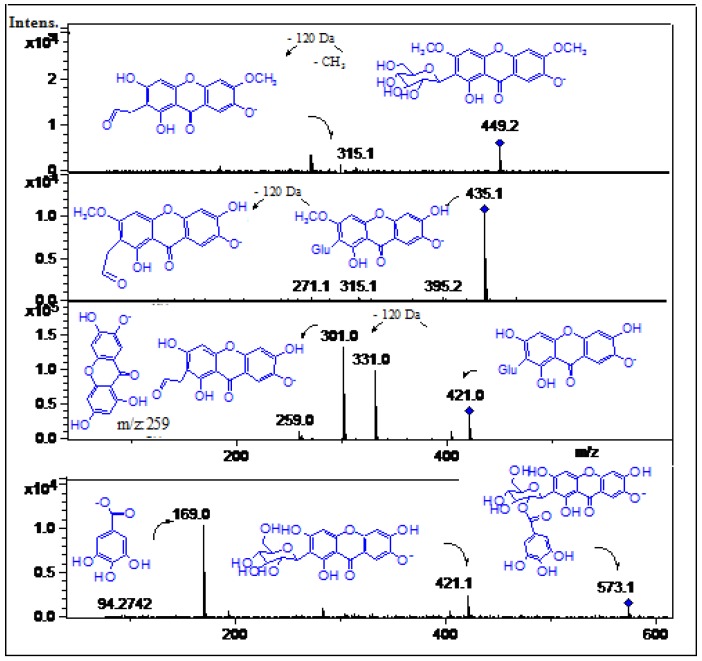
Structures, fragmentations, full ESI-MS and MS-MS spectra of peaks **10** and **12**–**14**.

In this work peaks **10**, **12**–**14** were tentatively identified as xanthones (Figure 3, Figure 4, Figure 5 and Figure 6). Mangosteen is one of the richest sources of different antioxidant xanthones [32], while mango fruits, specially the *Tommy* Atkins cultivar showed a few principal components, mainly mangiferin and isomangiferin [3,33]. Peak **12** showed an [M-H]^−^ ion at *m/z* 421.0 in the ToF-ESI-MS spectra and fragment ions at *m/z* 331.0 ([M-H-90 Daltons]^−^ and *m/z* 301.0 ([M-H-120 Daltons]^−^ corresponding to losses of C-glycoside phenolic compounds [21] and was identified as mangiferin [3], its identity being confirmed by spiking experiments using an authentic standard. Peak **14** with a pseudomolecular ion at *m/z* 449.2 and daughter ions at *m/z* 315.1 and *m/z* 301.0 was identified as the xanthone derivative di-methylmangiferin [30]. Peak **13** with a pseudomolecular ion at *m/z* 435.1 and MS^2^ ions at *m/z* 315.1, 301.0 and *m/z* 271.1 was identified as the mangiferin monomethyl derivative homomangiferin [29]. Similarly, Peak **10** with an [M-H]^−^ ion at *m/z* 573.1 and product MS^n^ ions at *m/z* 421.1 (mangiferin) and *m/z* 169.0 (gallic acid) was tentatively characterized as mangiferin gallate as reported [5].

#### 2.2.2. Phenolic Acids, tannins and their Derivatives and/or Related Compounds

**Figure 7:**
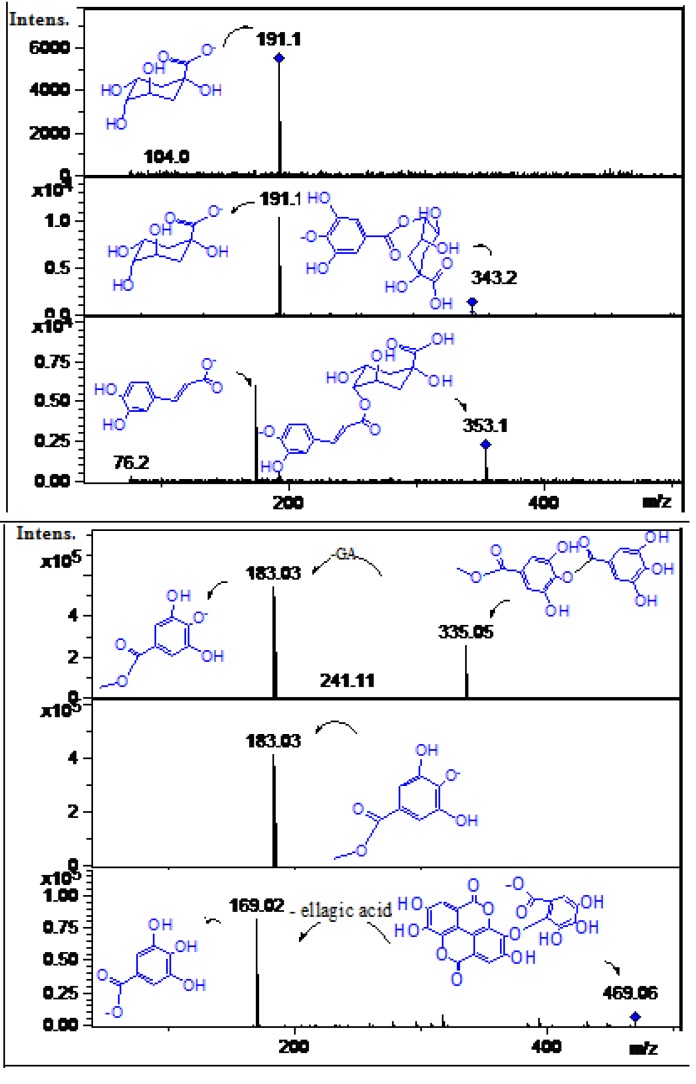
Structures, fragmentations, full ESI-MS and MS-MS spectra of peaks **1**, **2**, **3**, **4**, **5**, and **15**.

**Figure 8:**
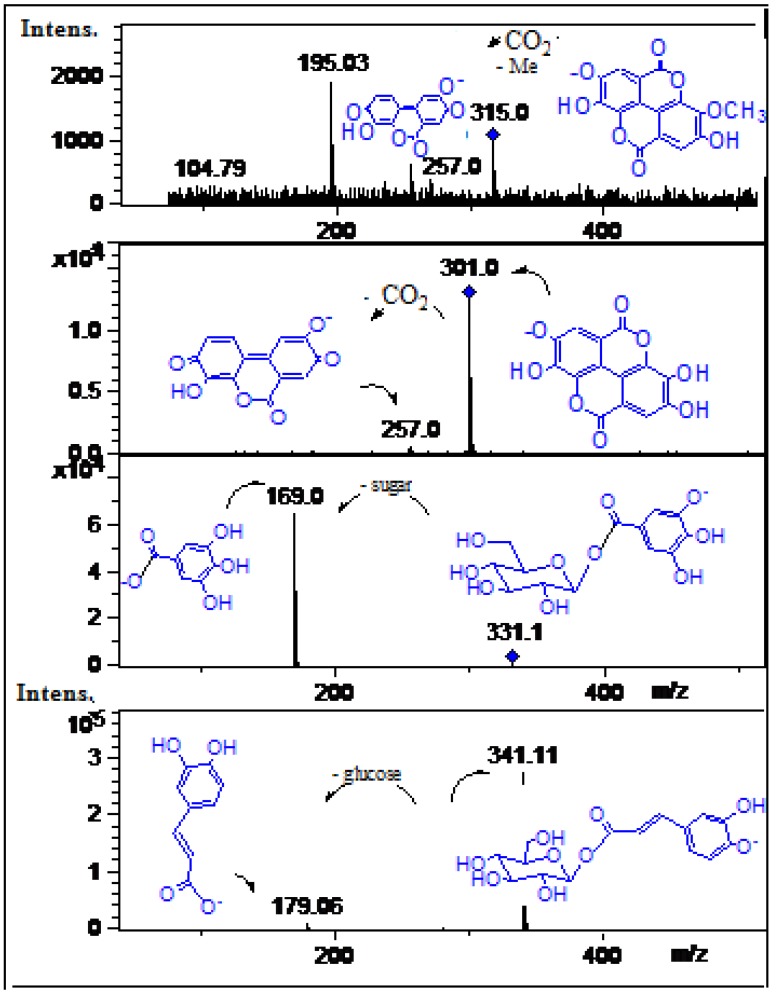
Structures, fragmentation, full ESI-MS and MS-MS spectra of peaks **8**, **11**, **20** and **21**.

Peak **1** was tentatively identified as quinic acid ([M-H]^−^ ion at *m/z* 191.1, Figure 7) while peak **15** with a [M-H]^−^ ion at *m/z* 343.1 and a loss of 152 Daltons (galloyl moiety) producing a quinic acid daughter ion at *m/z* 191.1 was a identified as galloylquinic acid (Figure 7) [22]. Peak **2** showed an [M-H]^−^ ion at *m/z* 335.2 and a daughter MS ion at *m/z* 183.0 (methyl gallate) and was identified as methyl digallate ester (Figure 7) while the related compound **4** was identified as methyl gallate (Figure 7) [22]. Peak **3** showing UV spectral data characteristic of an ellagic acid derivative (Figure 7 and Table 1) with an [M-H]^−^ ion at *m/z* 469.06 and a daughter gallic acid ion at *m/z* 169.02 was identified as the ellagic acid derivative valoneic acid bilactone (Figure 7) [23,24]. Peak **4** showed a pseudomolecular ion at *m/z* 183.03 and daughter MS ion at *m/z* 169.1 (gallic acid) and was identified as the gallic acid derivative methyl gallate [22]. Peak **8** with an [M-H]^−^ ion at *m/z* 331.0 and MS daughter ion at *m/z* 169.0 was identified as galloyl glucose (Figure 8) [27]. Peak **5** showed an [M-H]^−^ ion at *m/z* 353.1 (Figure 8) and an UV spectrum characteristic of caffeic acid (Table 1). A loss in the ESI MS-MS experiment resulting in a peak at *m/z* 179.0 (caffeic acid) prompted the identification of this compound as caffeoyl-quinic acid [25]. Similarly, peak **11** with an [M-H]^−^ ion at *m/z* 341.1 and a MS^2^ ion at *m/z* 179.6 was identified as caffeoylglucose (Figure 8) [25]. Peak **20** with a pseudomolecular anion at *m/z* 315.0 and daughter ions at *m/z* 257.0 and 195.03 was identified as methyl ellagic acid (Figure 8) [31]. Peak **21** with a pseudomolecular ion at *m/z* 301.0 and eluting very late in the chromatogram as reported [5] was identified as free ellagic acid (Figure 8) [5,20].

#### 2.2.3. Flavonoids

**Figure 9:**
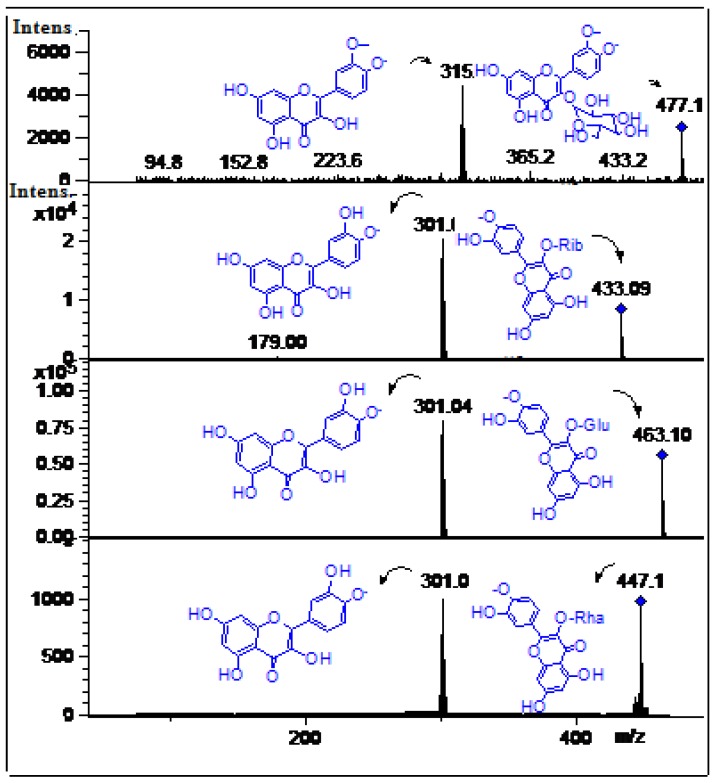
Structures, fragmentation, full ESI-MS and MS-MS spectra of peaks **16**, **17**, **18** and **19**.

Peak **16** showed an [M-H]^−^ ion at *m/z* 463.0 and a MS^2^ fragment at *m/z* 301.0 [6] which produced quercetin MS^3^ ions at *m/z* 179.0 and 151.0 [20] and was identified and confirmed as the flavonoid isoquercitrin (quercetin 3-*O*-glucoside, (Figure 9) [6], by spiking experiment with authentic standard. Similarly, peaks **17** and **18** with molecular ions at *m/z* 447.1 and 433.09 were identified as quercetin-3-*O*-pentoside and quercetin-3-*O*-rhamnoside respectively (Figure 9) [20]. Peak **19** with a molecular anion at *m/z* 477.1 and MS^2^ ion at *m/z* 315.1 (isorhamnetin) was identified as isorhamnetin 3-*O*-glucoside (Figure 9) [3].

#### 2.2.4. Procyanidins

**Figure 10:**
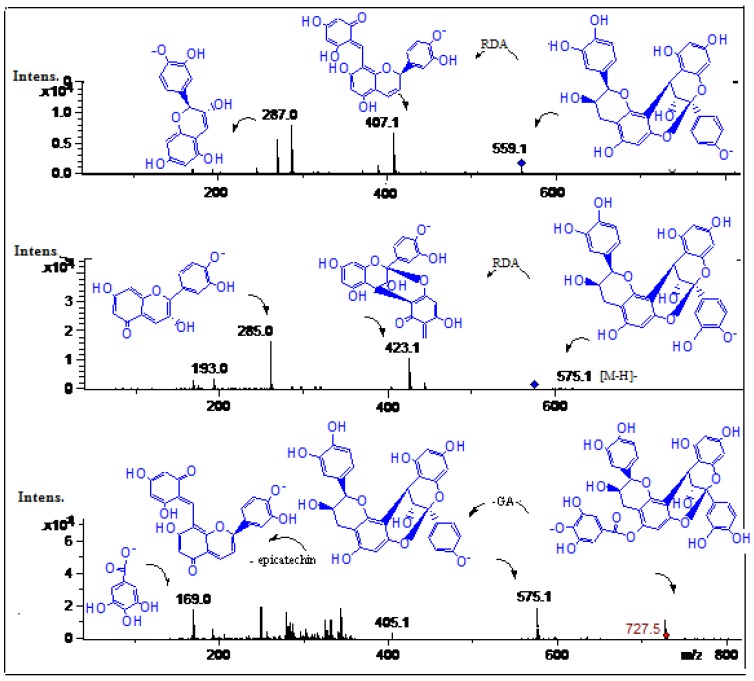
Structures, fragmentation, full ESI-MS and MS-MS spectra of peaks **6**, **7**, and **9**.

The monomer procyanidins (+)catechin and (−)epicatechin were identified in mango fruits in previous reports [17,34,35]. In this work minor peaks **6**, **7**, and **9** were identified as procyanidins (Figure 10). Peak **7** with an [M-H]^−^ ion at *m/z* 575.1 and product daughter ions at *m/z* 423.1 (RDA, Retro Diels Alder product) and 285.0 (epicatechin monomer) was identified as a procyanidin A dimer, (epi)catechin-(epi)catechin) [26]. Similarly, peak **9** with a molecular anion at *m/z* 559.1 and MS^2^ ions at *m/z* 407.1 product ion from RDA cleavage) and *m/z* 287.0 (epicatechin) was identified as a procyanidin A dimer with an epiafzelechin monomer constituent, (epiafzelechin-epicatechin) as reported [28]. Peak **6** with an [M-H]^−^ ion at *m/z* 727.5 and MS^n^ ions at *m/z* 405.1, 285.0 (epicatechin) and *m/z* 169.0 (gallic acid) was identified as a galloylated A type procyanidin dimer (galloyl-epicatechin-epicatechin) (Figure 10) [36].

### 2.3. Quantification of Phenolic Compounds

Phenolic compounds in peel and pulp of these two mango varieties were quantified using HPLC DAD and the results were compared (Table 2). Accuracy of the HPLC method was determined in recovery experiments, in which plant material was spiked with three different concentrations of standard compounds. As seen in Table 2 all recovery results were within the usually required recovery range of 100% ± 5%. Relative standard deviations were below 2.00% (Table 2) which indicated the methods repeatability [37]. Major compounds were **11** and **12** besides compounds **6**, **7**, **8** and **16** for all four plant parts studied while Pica peel (ppe) has the higher amount of compounds **11** (caffeoyl-glucose) and **12** (mangiferin), followed by *Tommy* peel (tpe, 35.43 ± 3.36 and 21.87 ± 4.45 for ppe and 24.57 ± 2.32 and 9.68 ± 1.83 for tpe, for compounds **11** and **12** respectively, Table 2). The amount of total phenolics quantified in this study (Table 2) were in the order *Pica* peel > *Tommy* peel > *Pica* pulp > *Tommy* pulp (66.02, 41.94, 29.89 and 29.37 mg/100 g fresh weight, respectively) the trend is similar to the trend found for total phenolics measured by the Folin Ciocalteau colorimetric method (Figure 2).

**Table 2:** Content of major phenolic compounds in peel and pulp of *Pica* and *Tommy* *Atkins* mango fruits.

Compound # (mg/100 g) ^a^	ppe	tpe	ppu	tpu	LOD-LOQ (ng/mL)	Recovery (mean ± RSD%)
**3**	0.52 ± 0.01	0.082 ± 0.00	0.12 ± 0.00	0.34 ± 0.01	nd	nd
**5**	0.83 ± 0.02	nd	0.02 ± 0.00	nd	nd	nd
**7**	2.87 ± 0.01	1.34 ± 0.00	1.97 ± 0.01	0.86 ± 0.45	nd	nd
**8**	3.76 ± 0.02	2.06 ± 0.01	1.44 ± 0.01	1.34 ± 0.02	nd	nd
**10**	0.91 ± 0.00	nd	2.35 ± 0.03	nd	nd	nd
**11**	34.13 ± 1.77	25.05 ± 1.67	14.90 ± 1.72	19.18 ± 1.12	nd	nd
**12**	22.15 ± 1.14	9.68 ± 0.64	4.24 ± 0.10	3.25 ± 0.1	12.3–41.6	99.52 ± 1.71
**13**	nd	nd	1.96 ± 0.01	1.71 ± 0.02	nd	nd
**14**	nd	nd	0.65 ± 0.01	nd	nd	nd
**15**	0.09 ± 0.00	nd	nd	nd	nd	nd
**16**	0.43 ± 0.02	3.12 ± 0.10	1.70 ± 0.04	2.66 ± 0.08	26.8–88.65	96.52 ± 0.99
**17**	nd	nd	0.42 ± 0.00	nd	nd	nd
**18**	nd	0.37 ± 0.00	0.052 ± 0.00	nd	nd	nd
**19**	0.30 ± 0.04a	0.24 ± 0.02a	0.027 ± 0.00b	0.031 ± 0.00b	23.37–73.4	102.48 ± 1.65
**20**	0.036 ± 0.00	nd	0.018 ± 0.00	nd	nd	nd
**21**	nd	nd	0.031 ± 0.00	nd	14.46-45.27	103.82 ± 1.24
**Total **	66.02	41.94	29.89	29.37	nd	nd

^a^ Expressed as mg/100 g fresh weight, measurements are expressed as mean ± SD of five parallel determinations. (Values in the same row marked with the same letter are not significantly different at *p* < 0.05). nd: not detected/determined.

## 3. Experimental

### 3.1. General

HPLC grade water, methanol, n-hexane, formic acid, HCl, KCl, Folin–Ciocalteu phenol reagent, sodium acetate, aluminum chloride hexahydrate and sodium carbonate were purchased from Merck (Darmstadt, Germany). Amberlite XAD-7HP 20-60 mesh resin, gallic acid, ellagic acid, quercetin, 1,1-diphenyl-2-picrylhydrazyl (DPPH) caffeic and gallic acids were purchased from Sigma Chemical Co. (St. Louis, MO, USA). Isorhamnetin-3-*O*-glucose, quercetin 3-*O*-glucose, the xanthone-*C*-glucoside mangiferin for HPLC analysis all with purity higher than 95% (with HPLC certificate) were purchased either from ChromaDex (Santa Ana, CA, USA) or Extrasynthèse (Genay, France). For the DPPH assay, a microplate reader spectrophotometer (Dynamica GmbH, Halo MPR-96, Zurich, Switzerland) was used. For the other spectroscopic measurements a Spectroquant Pharo UV-Vis spectrophotometer (Unico Instruments, Co, Ltd., Shanghai, China) was used.

### 3.2. Mango Cultivars

*Pica* and *Tommy Atkins* mango fruits were purchased at La Vega de Antofagasta. The fruits were collected in February 2012 in the first region of Chile (*Pica* mangoes from Pica, Tarapacá, Geographic coordinates: latitude 20°13'55.01"S; longitude 70°7'44.07"W, and *Tommy Atkins* mangoes from a plantation located in Valle de Azapa, Arica y Parinacota, Geographic coordinates*:* latitude: 18°31'34.93"S; longitude 70°9'58.94"W, Chile. Samples of the fruits are deposited at the laboratory of Natural products, University of Antofagasta with the numbers PiMa 150212 and ToMa 15022012.

### 3.3. Preparative Procedures

Pulps (100 g fresh material) and peels (100 g fresh material) from both mango cultivars were carefully washed and extracted (in triplicate) with MeOH (three times, 1 L each) in an ultrasonic bath in the dark at 25 °C per one hour each extraction. The methanolic extracts were filtered, combined and partitioned with n-hexane (three times, 1 L each) to remove lipids and waxes. The resulting MeOH extract were filtered, and concentrated *in vacuo* at 45 °C. The resulting MeOH extracts were suspended in water (25 mL) and loaded onto an Amberlite XAD-7 column (150 g) rinsed with water (1 L) and eluted with MeOH (1 L) to yield 2.38 ± 0.43 g of *Pica* mango pulp extract and 4.65 ± 0.67 g *Pica* mango peel extract, 1.67 ± 0.21 g of *Tommy Atkins* mango pulp extract and 4.48 ± 0.12 g *Tommy Atkins* mango peel extract, respectively.

### 3.4. HPLC DAD and HR-ESI-ToF-MS Conditions

An Agilent Series 1200 LC System (Agilent Technologies Inc., Waldbronn, Germany) coupled to a MicrOTOF Q II (Bruker Daltonics Inc., Billerica, MA, USA) was used for HPLC-HR-ESI-MS/MS analysis. The HPLC system consisted in a micro vacuum degasser, binary pumps, an autosampler (40 μL sample loop), a thermostated column compartment, and a diode array detector. The mass spectrometer equipped with electrospray ion source and qToF analyzer, was used in MS and MS/MS mode for the structural analysis of all compounds including flavonoids and phenolic acids. HPLC analyses were performed on a thermostated (40 °C) Phenomenex Luna C_18_ 250 × 4.6 mm (5 μm) column at 0.4 mL/min flow rate using 0.05% (v/v) formic acid (solvent A) and methanol (solvent B) with the following gradient of composition: starting with 20% solvent B and changing to 50% solvent B during 3 min, kept for 5 min, followed by a second ramp to 80% B in 5 min, maintained for 17 min, a third ramp to 20% B in 1 min, remaining at this last condition for 10 min before the next run ESI-MS detection was performed in negative and positive ion mode with mass acquisition between 100 and 1,500 Daltons. Nitrogen was used as drying and nebulizer gas (7 L/min and 3.5 Bar, respectively), and 180 °C for drying temperature. The injection volume was 10 μL. For MS/MS experiments fragmentation was achieved by using Auto MS^2^ option. PDA analyses were carried out in the range between 200 and 700 nm.

### 3.5. Polyphenolic Content

A precisely weighed amount of each extract (approximately 2 mg/mL) as explained in Section 3.3 was used for total phenolic (TPC) and total flavonoid (TFC) content. Extracts were dissolved in a MeOH:water 7:3 v/v solution. Appropriate dilutions were prepared and absorbance was measured using a spectrophotometer (see Section 3.1). The TPCs were determined by the Folin and Ciocalteu reagent method [38]. Briefly, the appropriate extract dilution was oxidized with the Folin-Ciocalteu reagent (2 mL, 10% v/v), and the reaction was neutralized with sodium carbonate. The calibration curve was performed with gallic acid (concentrations ranging from 16.0 to 500.0 μg/mL, R^2^ = 0.999). The absorbance of the resulting blue color of the complex formed was measured at 740 nm after 30 min, and the results were expressed as mg of gallic acid equivalents per g dry material. The TFCs in the samples were determined as previously reported [39,40]. The absorbance of the reaction mixture (2.5 mL) was measured at 430 nm and quercetin was used as a reference for the calibration curve (concentrations ranging from 16.0 to 800.0 µg/mL, R^2^ = 0.994). Results were expressed as mg quercetin equivalents per 100 g fresh weight. Data are reported as mean ± SD for at least three replications.

### 3.6. Antioxidant Assessment

#### 3.6.1. Bleaching of the 2,2-Diphenyl-1-picrylhydrazyl (DPPH) Radical Assay

Free radical scavenging capacity was evaluated using a microplate reader spectrophotometer according to the method described previously [41]. Briefly, aliquots of samples (50 μL, from 1.0 to 500 μg/mL) were assessed by their reactivity with a methanolic solution of 400 μM DPPH. The reaction mixtures (200 μL) were kept for 30 min at room temperature in the dark. The decreases in the absorbance (n = 3) were measured at 517 nm and the percentage of DPPH^●^ scavenging of the compounds and extracts was calculated using the following equation:

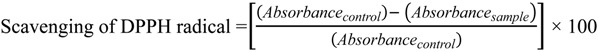

Afterwards, a curve of % DPPH scavenging activity versus concentration was plotted and IC_50_ values were calculated. IC_50_ denotes the concentration of sample required to scavenge 50% of DPPH free radicals. The lower the IC_50_ value the more powerful the antioxidant capacity. If IC_50_ ≤ 50 μg/mL the sample has high antioxidant capacity, if 50 μg/mL < IC_50_ ≤ 100 μg/mL the sample has moderate antioxidant capacity and if IC_50_ > 200 μg/mL the sample has no relevant antioxidant capacity. For comparison purposes, standard antioxidant compounds gallic acid (from 1.0 to 125.0 μg/mL, R^2^ = 0.991) and quercetin (from 1.0 to 125.0 μg/mL, R^2^ = 0.996) were used as standard antioxidant compounds, and were determined to have IC_50_ values of 11.87 ± 1.95 µg/mL (69.77 μmol/L) and 23.42 ± 3.12 µg/mL (77.48 μmol/L), respectively.

#### 3.6.2. Ferric Reducing Antioxidant Power (FRAP) Assay

The FRAP assay was performed according to [42] with some modifications. The stock solutions included 300 mM acetate buffer pH 3.6, 10 mM TPTZ (2,4,6-tripyridyl-*S*-triazine) solution in 40 mM HCl, and 20 mM FeCl_3_·6H_2_O solution. The working solution was prepared by mixing 50 mL acetate buffer, 10 mL TPTZ solution, and 15 mL FeCl_3_·6H_2_O solution and then warmed at 37 °C before using. Mango fruit extracts (100 μL) were allowed to react with 2 mL of the fresh FRAP solution for 30 min in the dark. Readings of the coloured product ferrous tripyridyltriazine complex were then taken at 593 nm (n = 3). The standard curve was performed with the standard antioxidant Trolox (R² = 0.998). Results are expressed in microMol TE (Trolox equivalents)/100 g fresh weight.

### 3.7. Quantitative Analysis

For quantitative analysis a standard stock solution was prepared by dissolving all standard compounds in 2.00 mL methanol (1.00 mg of compounds **12**, **16**, **19**, **21**). Five additional calibration levels were prepared by diluting this standard solution 1:2 with methanol to perform individual calibration curves (n = 5, r^2^ between 0.9961 and 0.9999). Due to the lack of all standard compounds, compound **3** (valoneic acid bilactone), **8** (galloyl glucose) and **15** (galloyl quinic acid) were quantified based on the calibration data of the structurally similar gallic acid, compound **5** and **11** were quantified using the calibration curve of caffeic acid, compounds **10**, **13** and **14** were quantified using calibration curve for **12** (mangiferin), Compounds **17** and **18** were quantified using calibration curve for **16** (quercetin 3-*O*-β-d-glucoside) and compound **20** was quantified using the calibration curve obtained for **21** (ellagic acid).

### 3.8. Method Validation

Method validation including linearity, precision and accuracy was carried out as recommended by [43]. The determined limits of detection (signal to noise ratio of three, based on a 10 μL injection) and limits of quantitation (S/N ratio of ten) were found to be below 27 ng/mL and 89 ng/mL, and indicated the sensitivity of the method employed. Accuracy was determined by spiking the sample with three concentrations of standard compounds (low, medium and high spike). For this purpose, known amounts of compounds **12**, **16**, **19** and **21** were added to a sample of the plant material (*Pica* peel), in triplicate which was then extracted (× 3 times) and assayed as described before. The measured amounts in relation to the theoretically present ones were expressed as percent of recovery and relative standard deviation was calculated (Table 2).

### 3.9. Statistical Analysis

The statistical analysis was carried out using the originPro 9.0 software packages (Originlab Corporation, Northampton, MA, USA). The determination was repeated at least five times for each sample solution. Analysis of variance was performed using one way ANOVA and Tukey test. (*p* values < 0.05 were regarded as significant).

## 4. Conclusions

Phenolic components of *Pica* mangoes were analyzed and quantified by HPLC for the first time. Furthermore, the extracts obtained from edible pulp of *Pica* mango fruits (local cultivar) showed moderate antioxidant capacity which is two times higher to that found for edible pulp of *Tommy Atkins* mango fruits cultivated in the same area of Chile. The antioxidant activity was high for both peels, which might be related with presence of diverse phenolic compounds and phenolic content found in these extracts. In this work 21 phenolic compounds were detected and tentatively characterized in mango fruits from northern Chile. Three procyanidin dimers (peaks **6**, **9** and **10**), seven acid derivatives (peaks **1**–**4**, **11**, **20** and **21**) and four xanthones were identified, mainly mangiferin (peak **12**) and mangiferin gallate, (peak **7**) which were present in both peel and pulp of the two species studied from northern Chile. Homomangiferin (peak **13**) was also present in both fruit pulps and di-methyl mangiferin (peak **14**) was present only in *Tommy* pulp. Four flavonoid glycosides were also identified, peaks **16**-**19**. Quercetin 3-*O*-glucoside (peak **16**) and isorhamnetin 3-*O* glucoside (peak **19**) were present in the four plant parts studied, while quercetin 3-*O*-rhamnoside (peak **17**) was present only in *Tommy* pulp and quercetin 3-*O*-pentoside (peak **18**) was present in *Pica* peel and *Tommy* pulp. The peel of *Pica* mangoes showed the highest content of phenolic compounds measured by HPLC-DAD where the two major compounds found were caffeoyl-glucose and mangiferin (34.13 ± 1.77 mg/100 g FW for compound **11** and 22.15 ± 1.14 mg/100 g FW for compound **12**, respectively). The HPLC fingerprints can be used to authenticate the mango cultivars and the compounds identified can be used as biomarkers for food products especially for *Mangifera indica* L*.* cv piqueño since little research has been reported for this species. *Mango de Pica* (*Mangifera indica* L*.* cv piqueño) can be considered as a rich source of important nutraceuticals, which are in higher amount than in regular commercial *Tommy Atkins* mango fruits.
